# Assessment of depression, PTSD, and insomnia symptoms in a cohort of Palestinian migrants residing in Jordanian camps during the outbreak of the War on Gaza: occurrence and correlates

**DOI:** 10.1007/s44192-024-00124-y

**Published:** 2025-01-27

**Authors:** Omar Gammoh, Saleh Bazi, Ruba Al Akash, Bilal Sayaheen, Mervat Alsous, Albara Alomari

**Affiliations:** 1 Department of Clinical Pharmacy and Pharmacy Practice, Yarmouk University, Shafiq Irshidat Str, P O Box 566, Irbid, 21163 Jordan; 2 Department of Marketing, Faculty of Business, Yarmouk University, Shafiq Irshidat Str., P O Box 566, Irbid, 21163 Jordan; 3 Refugees Displaced Persons and Forced Migration Studies Centre, Yarmouk University, Shafiq Irshidat Str., P O Box 566, Irbid, 21163 Jordan; 4 Faculty of Archaeology and Anthropology, Yarmouk University, Shafiq Irshidat Str., P O Box 566, Irbid, 21163 Jordan; 5 Department of Translation, Yarmouk University, Shafiq Irshidat Str, P O Box 566, Irbid, 21163 Jordan; 6 University of Doha for Science and Technology, Doha, Qatar

**Keywords:** War, Gaza, Mental health, Refugees

## Abstract

The present study sought to examine the occurrence and correlates of depression, PTSD, and insomnia in a cohort of Palestinian refugees residing in camps located in Jordan during the outbreak of the War on Gaza on Oct.7th.This is a cross-sectional cohort study that employed the convenient sampling method to recruit Palestinian refugees residing in Irbid and Azmi Almufti camps for Palestinian refugees. Depressive symptoms were assessed using the Patient Health Questionnaire (PHQ-9) scale. The self-reported PTSD symptoms were evaluated using the brief PTSD scale, and insomnia severity was assessed using the Insomnia Severity Index -Arabic scale (ISI-A). The data analysis from 258 participants showed that severe depression was reported in 178 participants (69%). Additionally, 127 participants (49.2%) reported severe PTSD symptoms, and 156 participants (60.5%) reported severe insomnia symptoms. Regression analysis revealed that “Residents of Azmi Almufti camp” was a significant correlate for severe depression (OR = 2.22, 95% 1.28–3.85, p = 0.004) and severe PTSD (OR = 1.81, 95% CI = 1.10–2.99, p = 0.02). The use of over-the-counter antihistamines as a sleep aid was a significant correlate severe insomnia (OR = 3.19, 95%CI = 1.17–8.71, p = 0.02) and PTSD (OR = 3.32, 95% CI = 1.34–8.21, p = 0.01). The conflict in Gaza has been observed to correlate with mental health challenges, particularly among Palestinian refugees residing in Jordan.

## Introduction

The existence of Palestinian refugees in Jordan is mainly linked to two major dates: 1948 and 1967. In 1948, a war erupted between Israel and the Arabs, resulting in the occupation of almost 77% of Palestinian land by Israel. This catastrophe is known in the Arab world as the Nakba. In 1967, another war erupted between Israel and the Arabs known as Al Naksa, ending with the occupation of new territories including, the Gaza Strip, Sinai Peninsula, the West Bank, and the Golan Heights. Besides the suffering and oppression of Palestinians that resulted from the two defeats, hundreds of thousands were forced to flee their homeland for neighboring countries such as, Lebanon, Syria and Jordan. The number of Palestinian refugee camps in Jordan is 10 official camps recognized by UNRWA (United Nations Relief and Works Agency), and 3 unofficial camps, namely the Prince Hassan Camp, the Madaba Camp, and the Sahab Camp. Palestinians residing in refugee camps are fragile economically and health wise, specifically those who reside in Azmi AlMofti Refugee Camp in Irbid. Moreover, The 35,000 Palestinian refugees residing in Azmi AlMofti Refugee Camp are confronting challenging conditions related to housing, education, humanitarian assistance, healthcare, and social welfare [1–3].

Mental health distress is known to be triggered or exacerbated by continuous exposure to horrific war scenes such as killing civilians, including women and children and bombarding houses, and hospitals [4]. The adverse impact on mental health stemming from exposure to certain content on social media and other communication resources primarily manifests as depression, post-traumatic stress disorder (PTSD), and insomnia. [5, 6].

Although continuous studies are highlighting the mental health challenges faced by Palestinians living in their homeland under occupation [7, 8], less evidence has examined the mental health status of the Palestinians residing in refugee camps in Jordan. According to the estimates, about 370,000 Palestinians live in refugee camps distributed in various Jordanian districts. This population is highly vulnerable to mental health distress due to the ongoing armed conflict in Palestine, their homeland, poverty, unemployment, and the challenging environment in their camps [9].

Therefore, the present study sought to examine the occurrence and correlates of depression, PTSD, and insomnia in a cohort of Palestinian refugees residing in camps located in Jordan, namely Irbid and Azmi Almufti camp located in Irbid, 80 km north of Amman, the capital, during the outbreak of the War on Gaza on October 7th.

## Material and methods

### Study design and settings

This is a cross-sectional cohort study that employed the convenient sampling method to recruit Palestinian refugees residing in Irbid and Azmi Almufti camps.

The study participants were enlisted through on-site visits. All procedures adhered to the principles outlined in the Declaration of Helsinki, and the research protocol was approved by XXXX University IRB committee. Informed consents were obtained from all participants. The research team communicated the study's objectives to potential participants through in-person interviews. Those who chose to participate were invited to sign a consent form; they retained the autonomy to discontinue their involvement at any stage. Data collection occurred in January 2024. A statistical G power calculation for logistic regression determined that a minimum of 250 participants were necessary for the study.

### Study instrument

The study instrument consisted of the covariates part and the outcome variable part. The covariate part comprised demographics and social media interaction concerning the war on Gaza. The outcome variables assessed were PTSD, depression, and insomnia symptoms.

### Covariates

This section crafted structured inquiries aimed at gathering demographic information from participants. Specifically, participants were asked to provide details regarding their age, gender, marital status, employment status, highest level of education attained, smoking habits, presence of chronic illnesses, and use of chronic medications. Participants were asked whether they have relatives or friends residing in Gaza and how they overcome their sleeping problem by using over-the-counter (OTC) sedating antihistamines as sleep aids or household homeopathic relaxing remedies. Additionally, participants were asked about the frequency with which they share news related to the Gaza war and their opinions on temporarily abstaining from social media interactions to mitigate potential mental health impacts.

### Outcome variables

#### Depression symptoms

The self-reported depressive symptoms were assessed through the Patient Health Quality-9 scale (PHQ-9) Arabic version. This validated scale provides a comprehensive capture of depression symptoms through nine questions rated against Likert-type answers. A score above 14 indicates severe depression. [10, 11]

#### Post-traumatic stress disorder symptoms

The self-reported PTSD symptoms were evaluated using the brief PTSD scale originally developed by Foa et al. 1993, translated to Arabic, and validated by [12] with a Cronbach alpha of 0.79. The scale consists of 17 questions to capture PTSD symptoms according to the DSM-IV guidelines. A score above 25 on this scale reflects severe PTSD symptoms.

#### Insomnia symptoms

The assessment of the self-reported insomnia burden was conducted through the Insomnia Severity Index Arabic version (ISI-A). This validated and reliable scale (Cronbach alpha = 0.81) comprises 7 questions that explore sleep quality for the past week, with a total score range between 0 and 28. A score exceeding 14 indicates severe insomnia. [13, 14]

### Data analysis

Data were analyzed using SPSS software version 21. The demographic information of the participants was expressed as frequencies and percentages. Three outcome variables (dependent variables) were found in the study: depression, PTSD, and insomnia. Therefore, three distinct binary regression analyses were carried out. Initially, to identify the potential confounders, a univariate analysis was performed to identify the potential confounders showing p < 0.1, which were used to feed the multivariable binary regression model using the backward stepwise approach to identify which factors are independently associated with each of the outcome variables. A p-value of less than 0.05 was considered to be statistically significant.

## Results

### Participants characteristics

The data analysis from 258 participants showed that 166 (64.3%) were males, 173 (67%) were aged above 26 years, 137(53%) were married, 133 (51.5%) were residents of Irbid camp, 160 (62.01%) were employed, 83 (31.9%) reported having relatives or friends in Gaza, 45 (17.44%) reported losing a family member or a friend in Gaza, 29 (11.2%) reported using self-medication with OTC sedating antihistamine to improve sleep, and 16 (6.2%) reported using self-medication with herbal homeopathy to improve sleep. Table 1.

**Table 1:** The demographical information of the study sample (n = 258)

Factor	Category	(n; %)
Gender	Male	(166; 64.3%)
	Female	(92; 35.7%)
Age	Below 26 years	(85; 33%)
	26 years & above	(173; 67%)
Marital Status	Single	(121; 47%)
	Married	(137; 53%)
Palestinians Camp location	Irbid Camp	(133; 51.5%)
	Maryr Azmi Al-Mufti Camp	(125; 48.5%)
Employment Status	Unemployed	(98; 37.9%)
	Employed	(160; 62.01%)
Smoking	Non-smoking	(124; 48.1%)
	Smoking	(134; 51.9%)
Relative and Friends in Gaza	Yes, I have	(83; 31.9%)
	No, I don’t have	(175; 68.1%)
Was one of your relatives or acquaintances martyred in Gaza in the current aggression?	Yes	(45; 17.44%)
	No	(188; 72.87%)
	I lost communication	(25; 9.69%)
How are you trying to overcome your current sleep problem? I don't have a problem sleeping	Self-medication with OTC sedating antihistamine to improve my sleep	(29; 11.2%)
	Self- medication with herbal homeopathy to improve my sleep	(16; 6.2%)
Diagnosed with chronic diseases	No	(217;84.1%)
	Yes	(40; 15.5%)
Receiving long-term medications	No	(208; 80.6%)
	Yes	(45; 17.4%)
How often do you post content (e.g., status updates, photos, videos) to your social media accounts?	Several times a day daily	(75; 29.1%)
	2–3 times a week	(47; 18.2%)
	Once a week	(27; 10.5%)
	Rarely	(108; 42.8%)
Have you ever taken a break or deactivated your social media accounts due to concerns about your health or safety?	Yes	(89; 34.5%)
	No	(169; 65.5%)

### Depression: occurrence and correlates

The severity of depression was assessed through the PHQ-9 scale Arabic version, participants scoring 15 and above were considered severely depressed. Therefore, our analysis revealed that 178 participants (69%) reported severe depression. To identify the factors that could be potentially associated with severe depression, a univariate analysis was conducted. The potential confounders: “age”, “marital status”, resident of Azmi Almufti camp”, “employment”, “using OTC antihistamines as a sleep aid”; and long-term medications were identified and used to feed the subsequent multivariable model, which was performed using the backward stepwise model. The multivariable model finally included “resident of Azmi Almufti camp” as a significant correlate for severe depression (OR = 2.22, 95% 1.28–3.85, p = 0.004). Table 2

**Table 2:** The association between the participants’ covariates and severe depression (according to the PHQ-9 scale) using univariate and multivariable analysis

Factor	Univariate analysis			Multivariable analysis		
	OR	95% CI	p	OR	95% CI	p
Gender	0.89	0.52–1.54	0.68			
Age	1.57	0.91–2.72	0.10			
Marital Status	1.72	1.01–2.94	0.04*			
Azmi Almufti Camp residents	2.22	1.28–3.84	0.004*	2.22	1.28–3.84	0.004
Employment Status	1.78	1.04–3.04	0.04*			
Smoking	1.49	0.88–2.54	0.13			
Relative and Friends in Gaza	0.86	0.49–1.53	0.61			
How often do you post content (e.g., status updates, photos, videos) to your social media accounts?	0.86	0.69–1.05	0.14			
Have you ever taken a break or deactivated your social media accounts due to concerns about your health or safety?	1.04	0.74–1.49	0.80			
Self-medication with OTC sedating antihistamine to improve my sleep	2.82	0.94–8.44	0.06			
Self- medication with herbal homeopathy to improve my sleep	3.07	0.67–13.95	0.14			
Diagnosed with chronic diseases	1.68	0.75–3.71	0.20			
Receiving long-term medications	2.17	0.98–4.82	0.05			

### PTSD: occurrence and correlates

We used the Arabic-validated Scale [12] for assessing the self-reported PTSD symptoms with a cut-off score above 25 for severe PTSD symptoms. We report that 127 participants (49.2%) reported severe PTSD symptoms. The univariate analysis was carried out to identify the potential confounders of severe PTSD. We report that “smoking”, “Resident of Azmi Almufti camp”, “posting the war news on social media”, and “using OTC antihistamines as a sleep aid” were regarded as potential confounders and were further used to build the multivariable binary logistic regression model as above. The multivariable model finally included “Resident of Azmi Almufti camp”, and “using OTC antihistamines as a sleep aid” as significant correlate for severe PTSD (OR = 1.81, 95% CI = 1.10–2.99, p = 0.02) and (OR = 3.32, 95% CI = 1.34–8.21, p = 0.01) respectively. Table 3.

**Table 3:** The association between the participants’ covariates and severe PTSD (according to the Foa scale) using univariate and multivariable analysis

Factor	Univariate analysis			Multivariable analysis		
	OR	95% CI	p	aOR	95% CI	p
Gender	0.98	0.59–1.63	0.94			
Age	1.41	0.83–2.37	0.20			
Marital Status	1.33	0.81–2.17	0.26			
Azmi Almufti Camp residents	1.81	1.1–2.96	0.01*	1.81	1.10–2.99	0.02*
Employment Status	1.16	0.70–1.92	0.57			
Smoking	1.76	1.07–2.88	0.02*			
Relative and Friends in Gaza	0.80	0.47–1.35	0.40			
How often do you post content (e.g., status updates, photos, videos) to your social media accounts?	0.83	0.68–1.00	0.05			
Have you ever taken a break or deactivated your social media accounts due to concerns about your health or safety?	0.82	0.59–1.14	0.25			
Self-medication with OTC sedating antihistamines to improve my sleep	3.31	1.35–8.13	0.009*	3.32	1.34–8.21	0.01*
Self- medication with herbal homeopathy to improve my sleep	2.15	0.71–6.48	0.17			
Diagnosed with chronic diseases	0.81	0.41–1.59	0.54			
Receiving long-term medications	1.03	0.54–1.96	0.91			

### Insomnia: occurrence and correlates

Insomnia was assessed using the ISI-A scale, with a threshold score above 14 used to screen for severe insomnia symptoms. A total of 156 participants (60.5%) reported severe insomnia symptoms. The univariate analysis identified “receiving long-term medications for chronic diseases” and “using OTC antihistamines as a sleep aid” as potential confounders. The multivariable binary logistic regression showed that only using OTC antihistamines as a sleep aid” was a correlate for severe insomnia (OR = 3.19, 95%CI = 1.17–8.71, p = 0.02). Table 4.

**Table 4:** The association between the participants’ covariates and severe insomnia (according to the ISI-A scale) using univariate and multivariable analysis

Factor	Univariate analysis			Multivariable analysis		
	OR	95% CI	p	aOR	95% CI	p
Gender	1.18	0.70–2.00	0.52			
Age	1.28	0.75–2.17	0.36			
Marital Status	1.23	0.74–2.02	0.42			
Azmi Almufti Camp residents	1.03	0.62–1.69	0.92			
Employment Status	1.33	0.80–2.23	0.26			
Smoking	1.13	0.69–1.87	0.61			
Relative and Friends in Gaza	1.09	0.64–1.86	0.74			
How often do you post content (e.g., status updates, photos, videos) to your social media accounts?	1.10	0.89–1.36	0.35			
Have you ever taken a break or deactivated your social media accounts due to concerns about your health or safety?	1.01	0.72–1.41	0.94			
Self-medication with OTC sedating antihistamines to improve my sleep	3.19	1.17–8.71	0.02*	3.19	1.17–8.71	0.02*
Self- medication with herbal homeopathy to improve my sleep	1.86	0.57–6.01	0.30			
Diagnosed with chronic diseases	1.65	0.79–3.24	0.17			
Receiving long-term medications	1.72	0.86–3.44	0.10			

## Discussion

The present study aimed to identify the occurrence and correlates of depression, PTSD, and insomnia as self-reported symptoms in a cohort of Palestinian refugees residing in Jordan during the outbreak of the War on Gaza. We report a high rate of all the above-mentioned symptoms. Also, our findings revealed that the residents of Azmi Almufti camp were at higher risk for severe depression and PTSD symptoms. In addition, using sedating antihistamines as a sleep aid was associated with severe PTSD and insomnia symptoms.

This huge burden of depression and PTSD in the study sample is attributed to multifactorial, interrelated, and complicated factors, a nexus worth exploring. Our findings demonstrated that the refugees residing in Azmi Almufti camp were more prone to severe depression and PTSD. Exploring mental health status in this population is faced with social and cultural barriers [9]. Poor living conditions, poverty, and a lack of jobs could explain, at least in part, this finding. According to published data dated back in 2011, Azmi Almufti camp is the second poorest camp with the highest rate of unemployability in general and in women reaching up to 18% in total and 25% for females [15]. Although no updates are yet available about the socio-economic status of the Azmi Almufti camp, however, the authors expect a more challenging situation due to the Syrian conflict that has led to Syrian refugees migration mainly to Irbid, thus posing additional restrains on Jordan, the hosting country.

Poor mental health status encompasses the behavior of individuals to adopt negative behaviors, for example, in a cross-sectional study comprising more than 73,000 Palestinians living in Jordan camps, depressive symptoms were associated with days of missing schools, the same study points out the high rates of depressive symptoms namely loneliness, suicidal ideation, and worry among adolescents [16].

The findings of this study highlighted that participants using OTC sedating antihistamines as a sleep aid were at higher association for severe insomnia and PTSD symptoms. Self-medication with sedating antihistamines is a common practice in many populations due to its availability, safety, and affordability [17]. In addition, earlier, our research group demonstrated that OTC sedating antihistamines are the most recommended sleep aid by community pharmacists in Jordan [18]. Our findings can be explained as follows: participants experiencing severe insomnia and PTSD symptoms tend to use the “available” medications to alleviate their symptoms, conversely, these severe symptoms could be due to antihistamines, as they stimulate the Central Nervous System, which could result in difficulty sleeping or insomnia or users may take overdoses of antihistamines due to tolerance [19]. According to the literature, the clear benefits of sedating antihistamines have not been proven yet [20, 21]. Similarly, antihistamines are ineffective in alleviating PTSD symptoms. Furthermore, they could exacerbate symptoms due to their complex pharmacological actions [22]. This is the first study, to the best our knowledge, that highlighted the occurrence and correlates of depression, PTSD, and insomnia experienced by the Palestinian refugees residing in Jordan during the outbreak of the October7^th^ war in their homeland in Palestine- Gaza. Although the current study has several strengths, including the unique sample type, the validated scales, and the sound data analysis performed, some limitations exist. For example, the study did not include a follow-up phase to monitor the changes in the mental health spectrum of the participants, also, the study did not provide a thorough psychiatric assessment by specialists in the camps due to social and cultural barriers to mental health.

## Conclusion

In conclusion, the impact of the ongoing war on Gaza demonstrated to be associated with mental health distress outside the borders of Gaza. Palestinian refugees residing in Jordan for decades are highly affected mentally. Comprehensive and immediate actions are warranted to address the mental health and well-being of this vulnerable community by addressing the modifiable correlating factors.

## Data Availability

The data will be provided by the corresponding author upon request.
